# Supplementary material for: Identifying agricultural disaster risk zones for future climate actions
Source: PLoS One. 2021 Dec 2;16(12):e0260430. doi: 10.1371/journal.pone.0260430 (PMC8638849; doi:10.1371/journal.pone.0260430)
Supplement: S1 File — (PDF) [file pone.0260430.s001.pdf]

Supporting information

Table 4. List of all crops covered by the FAO and used in this study

		CROPS		
Agave fibres	Almonds	Anise, coriander	Apples	Apricots
Areca nuts	Artichokes	Asparagus	Avocados	Bambara beans
Bananas	Barley	Bast fibres	Beans, dry	Beans, green
Berries nes	Blueberries	Brazil nuts	Broad and horse beans	Buckwheat
Cabbages	Canary seed	Carobs	Carrots	Cashew nuts
Cashew apple	Cassava	Cassava leaves	Castor oil seed	Cauliflowers & broccoli
Cereals nes	Cherries	Cherries, sour	Chestnut	Chick peas
Chicory roots	Peppers, dry	Peppers, green	Cinnamon (cannella)	Cloves
Cocoa, beans	Coconuts	Coffee, green	Coir	Cow peas, dry
Cranberries	Cucumbers and gherkins	Currants	Dates	Eggplants (aubergines)
Fibre crops nes	Figs	Flax fibre and tow	Fonio	Fruit, citrus
Fruit, fresh	Fruit, pome	Fruit, stone	Fruit, tropical	Garlic
Ginger	Gooseberries	Grain, mixed	Grapefruit	Grapes
Groundnuts	Gums, natural	Hazelnuts	Hemp tow waste	Hemp seed
Hops	Jojoba seed	Jute	Kapok fruit	Karite nuts
Kiwi fruit	Kola nuts	Leeks	Lemons and limes	Lentils
Lettuce and chicory	Linseed	Lupins	Maize	Maize, green
Mangoes, guavas	Manila fibre	Maté	Melons	Melon seed
Millet	Mushrooms and truffles	Mustard seed	Nutmeg, cardamoms	Nuts
Oats	Oil palm fruit	Oil seeds	Okra	Olives
Onions, dry	Onions, green	Oranges	Papayas	Peaches and nectarines
Pears	Peas, dry	Peas, green	Pepper	Peppermint
Persimmons	Pigeon peas	Pineapples	Pistachios	Plantains
Plums and sloes	Poppy seed	Potatoes	Pulses nes	Pumpkins
Pyrethrum, dried	Quinces	Quinoa	Ramie	Rapeseed
Raspberries	Rice, paddy	Rice, paddy (RME)	Roots and tubers	Rubber, natural
Rye	Safflower seed	Seed cotton	Sesame seed	Sisal
Sorghum	Soybeans	Spices nes	Spinach	Strawberries
String beans	Sugar beet	Sugar cane	Sugar crops nes	Sunflower seed
Sweet potatoes	Tallow tree seed	Tangerines, mandarins	Taro (cocoyam)	Tea
Tobacco	Tomatoes	Triticale	Tung nuts	Vanilla
Vegetables, fresh	Vegetables, leguminous	Vetches	Walnuts	Watermelons
Wheat	Yams	Yautia (cocoyam)		

Table 5. List of livestock primary covered by the FAO and used in this study

	LIVESTOCK PRIMARY		
Beeswax	Eggs, hen, in shell	Eggs, hen, in shell (number)	Eggs, other bird, in shell
Eggs, other bird, in shell (number)	Fat, buffaloes	Fat, camels	Fat, cattle
Fat, goats	Fat, pigs	Fat, sheep	Hides, buffalo, fresh
Hides, cattle, fresh	Honey, natural	Meat nes	Meat, ass
Meat, bird nes	Meat, buffalo	Meat, camel	Meat, cattle
Meat, chicken	Meat, duck	Meat, game	Meat, goat
Meat, goose and guinea fowl	Meat, horse	Meat, mule	Meat, other camelids
Meat, other rodents	Meat, pig	Meat, rabbit	Meat, sheep
Meat, turkey	Milk, whole fresh buffalo	Milk, whole fresh camel	Milk, whole fresh cow
Milk, whole fresh goat	Milk, whole fresh sheep	Offals, edible, buffaloes	Offals, edible, camels
Offals, edible, cattle	Offals, edible, goats	Offals, horses	Offals, pigs, edible
Offals, sheep,edible	Silk-worm cocoons, reelable	Skins, goat, fresh	Skins, sheep, fresh
Snails, not sea	Wool, greasy		

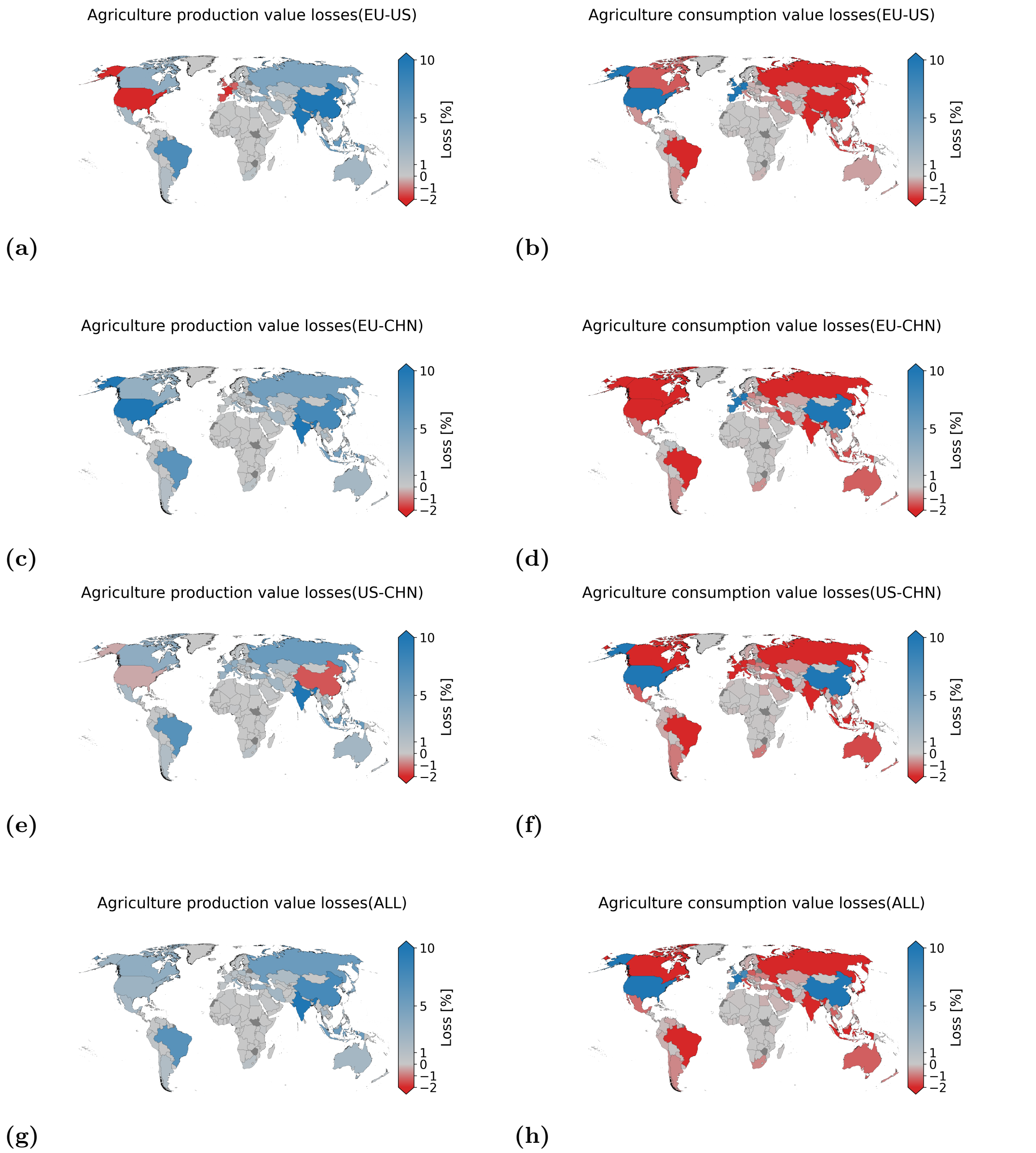

Fig 7. Share of agricultural production and consumption value losses when concurrent extreme weather events hit two or more breadbasket regions. (a), (c), (e), and (g) are the production value losses when the extreme event hit the EU and US (EU-US), EU and China (EU-CHN), US and China (US-CHN), and All three regions (ALL) respectively. (b), (d), (f), and (h) are their corresponding consumption value losses. Since we are computing the losses, negative values(red) implies a rise in production/consumption value while positive values implies a drop in production/consumption value (drop). Values are in billion USD. This figure was generated with the Mapping package in python.

Table 6. Country names, their ISO3 codes and continents used in Figures 5, 6 and 7

ISO3	Country name	Continent
AFG	Afghanistan	Asia
ALB	Albania	Europe
DZA	Algeria	Africa
AND	Andorra	Europe
AGO	Angola	Africa
ATG	Antigua and Barbuda	N. America
ARG	Argentina	S. America
ARM	Armenia	Asia
ABW	Aruba	S. America
AUS	Australia	Oceania
AUT	Austria	Europe
AZE	Azerbaijan	Asia
BHS	Bahamas	N. America
BHR	Bahrain	Asia
BGD	Bangladesh	Asia
BRB	Barbados	N. America
BLR	Belarus	Europe
BEL	Belgium	Europe
BLZ	Belize	N. America
BEN	Benin	Africa
BMU	Bermuda	N. America
BTN	Bhutan	Asia
BOL	Bolivia	S. America
BIH	Bosnia and Herzegovina	Europe
BWA	Botswana	Africa
BRA	Brazil	S. America
VGB	British Virgin Islands	S. America
BRN	Brunei Darussalam	Asia
BGR	Bulgaria	Europe
BFA	Burkina Faso	Africa
BDI	Burundi	Africa
KHM	Cambodia	Asia
CMR	Cameroon	Africa
CAN	Canada	N. America
CPV	Cabo Verde	Africa
CYM	Cayman Islands	S. America
CAF	Central African Republic	Africa
TCD	Chad	Africa
CHL	Chile	S. America
CHN	China	Asia
COL	Colombia	S. America
COG	Republic Congo	Africa
CRI	Costa Rica	N. America
HRV	Croatia	Europe
CUB	Cuba	N. America
CYP	Cyprus	Europe
CZE	Czech Republic	Europe
CIV	Côte d'Ivoire	Africa
PRK	North Korea	Asia
COD	Democratic Republic Congo	Africa
DNK	Denmark	Europe
DJI	Djibouti	Africa
DOM	Dominican Republic	N. America
ECU	Ecuador	S. America
EGY	Egypt	Africa
SLV	El Salvador	N. America

Table 7. Country names, their ISO3 codes and continents used in Figures 5, 6 and 7

ISO3	Country name	Continent
ERI	Eritrea	Africa
EST	Estonia	Europe
ETH	Ethiopia	Africa
FJI	Fiji	Oceania
FIN	Finland	Europe
FRA	France	Europe
PYF	French Polynesia	Oceania
GAB	Gabon	Africa
GMB	Gambia	Africa
GEO	Georgia	Asia
DEU	Germany	Europe
GHA	Ghana	Africa
GRC	Greece	Europe
GRL	Greenland	N. America
GTM	Guatemala	N. America
GIN	Guinea	Africa
GUY	Guyana	S. America
HTI	Haiti	N. America
HND	Honduras	N. America
HKG	Hong Kong	Asia
HUN	Hungary	Europe
ISL	Iceland	Europe
IND	India	Asia
IDN	Indonesia	Asia
IRN	Iran	Asia
IRQ	Iraq	Asia
IRL	Ireland	Europe
ISR	Israel	Asia
ITA	Italy	Europe
JAM	Jamaica	N. America
JPN	Japan	Asia
JOR	Jordan	Asia
KAZ	Kazakhstan	Asia
KEN	Kenya	Africa
KWT	Kuwait	Asia
KGZ	Kyrgyz Republic	Asia
LAO	Lao PDR	Asia
LVA	Latvia	Europe
LBN	Lebanon	Asia
LSO	Lesotho	Africa
LBR	Liberia	Africa
LBY	Libya	Africa
LIE	Liechtenstein	Europe
LTU	Lithuania	Europe
LUX	Luxembourg	Europe
MAC	Macao	Asia
MDG	Madagascar	Africa
MWI	Malawi	Africa
MYS	Malaysia	Asia
MDV	Maldives	Asia
MLI	Mali	Africa
MLT	Malta	Europe
MRT	Mauritania	Africa
MUS	Mauritius	Africa
MEX	Mexico	N. America
MCO	Monaco	Europe

Table 8. Country names, their ISO3 codes and continents used in Figures 5, 6 and 7

ISO3	Country name	Continent
MNG	Mongolia	Asia
MNE	Montenegro	Europe
MAR	Morocco	Africa
MOZ	Mozambique	Africa
MMR	Myanmar	Asia
NAM	Namibia	Africa
NPL	Nepal	Asia
NLD	Netherlands	Europe
ANT	Netherlands Antilles	Europe
NCL	New Caledonia	Oceania
NZL	New Zealand	Oceania
NIC	Nicaragua	N. America
NER	Niger	Africa
NGA	Nigeria	Africa
NOR	Norway	Europe
PSE	West Bank and Gaza	Asia
OMN	Oman	Asia
PAK	Pakistan	Asia
PAN	Panama	S. America
PNG	Papua New Guinea	Asia
PRY	Paraguay	S. America
PER	Peru	S. America
PHL	Philippines	Asia
POL	Poland	Europe
PRT	Portugal	Europe
QAT	Qatar	Asia
KOR	South Korea	Asia
MDA	Moldova	Europe
ROU	Romania	Europe
RUS	Russian Federation	Asia
RWA	Rwanda	Africa
WSM	Samoa	Oceania
SMR	San Marino	Europe
STP	São Tomé and Principe	Africa
SAU	Saudi Arabia	Asia
SEN	Senegal	Africa
SRB	Serbia	Europe
SYC	Seychelles	Africa
SLE	Sierra Leone	Africa
SGP	Singapore	Asia
SVK	Slovak Republic	Europe
SVN	Slovenia	Europe
SOM	Somalia	Africa
ZAF	South Africa	Africa
SSD	South Sudan	Africa
ESP	Spain	Europe
LKA	Sri Lanka	Asia
SDN	Sudan	Africa
SUR	Suriname	S. America
SWZ	Swaziland	Africa
SWE	Sweden	Europe
CHE	Switzerland	Europe
SYR	Syrian Arab Republic	Asia
TWN	Taiwan	Asia
TJK	Tajikistan	Asia
THA	Thailand	Asia

Table 9. Country names, their ISO3 codes and continents used in Figures 5, 6 and 7

ISO3	Country name	Continent
MKD	Macedonia	Europe
TGO	Togo	Africa
TTO	Trinidad and Tobago	S. America
TUN	Tunisia	Africa
TUR	Turkey	Asia
TKM	Turkmenistan	Asia
UGA	Uganda	Africa
UKR	Ukraine	Europe
ARE	United Arab Emirates	Asia
GBR	United Kingdom	Europe
TZA	Tanzania	Africa
USA	United States of America	N. America
URY	Uruguay	S. America
UZB	Uzbekistan	Asia
VUT	Vanuatu	Oceania
VEN	Venezuela	S. America
VNM	Vietnam	Asia
YEM	Yemen	Asia
ZMB	Zambia	Africa
ZWE	Zimbabwe	Africa
