# Supplementary material for: Identifying agricultural disaster risk zones for future climate actions
Source: PLoS One. 2021 Dec 2;16(12):e0260430. doi: 10.1371/journal.pone.0260430 (PMC8638849; doi:10.1371/journal.pone.0260430)
Supplement: Attachment — Submitted filename: PlosOne_Response_to_Reviewer2.doc [file pone.0260430.s002.doc]

**Response to Reviewer 2**

This is an interesting article presenting methodology to identify agricultural disaster risk zones which is vital to combat possible climate change impacts in future. The article is well written, it can be considered for publication after addressing the following comments.

Response: Thank you very much for your complements and your suggestions have helped to improve the content of the paper.

Abstract: Well written – no comments.

Response: The abstract has been restructured for a better understanding.

Introduction: The introduction is very good, the authors demonstrate a thorough knowledge of the published literature and highlight the importance and background to carry out this investigation

Response: Thank you.

Methods: Methods are technically strong and well explained.

Response: Thank you.

Results and Discussion: Results are well explained. However, the discussion concerning other published papers on the topic must be included.

Response: A new section on discussion has been added and the discussions about how the results here tie to other results have been included. Thank you.

Fig 2, The legend in the sub-figures do not make much sense. The author may try to elaborate on the regions in fig 2(a) and the abbreviations in figures 2b-d.

Response: The legends were wrong and have been updated. New figures have been added here for clarification. The ISO3 codes were used in the place of country names. A list of country names and their ISO3 codes have been added in the supplementary section for a better comprehension.

Conclusion: No Comment.

Response: This section has equally been updated. Thank you.
